# Emerging concepts involving inhibitory and activating RNA functionalization towards the understanding of microcephaly phenotypes and brain diseases in humans

**DOI:** 10.3389/fcell.2023.1168072

**Published:** 2023-06-20

**Authors:** Mayuri Tokunaga, Takuya Imamura

**Affiliations:** Program of Biomedical Science, Graduate School of Integrated Sciences for Life, Hiroshima University, Hiroshima, Japan

**Keywords:** microcephaly, non-coding RNA, RNA-binding protein, human medicine, epigenetic, brain diversity

## Abstract

Microcephaly is characterized as a small head circumference, and is often accompanied by developmental disorders. Several candidate risk genes for this disease have been described, and mutations in non-coding regions are occasionally found in patients with microcephaly. Various non-coding RNAs (ncRNAs), such as microRNAs (miRNAs), SINEUPs, telomerase RNA component (TERC), and promoter-associated lncRNAs (pancRNAs) are now being characterized. These ncRNAs regulate gene expression, enzyme activity, telomere length, and chromatin structure through RNA binding proteins (RBPs)-RNA interaction. Elucidating the potential roles of ncRNA-protein coordination in microcephaly pathogenesis might contribute to its prevention or recovery. Here, we introduce several syndromes whose clinical features include microcephaly. In particular, we focus on syndromes for which ncRNAs or genes that interact with ncRNAs may play roles. We discuss the possibility that the huge ncRNA field will provide possible new therapeutic approaches for microcephaly and also reveal clues about the factors enabling the evolutionary acquisition of the human-specific “large brain.”

## 1 Introduction

**FIGURE 1:**
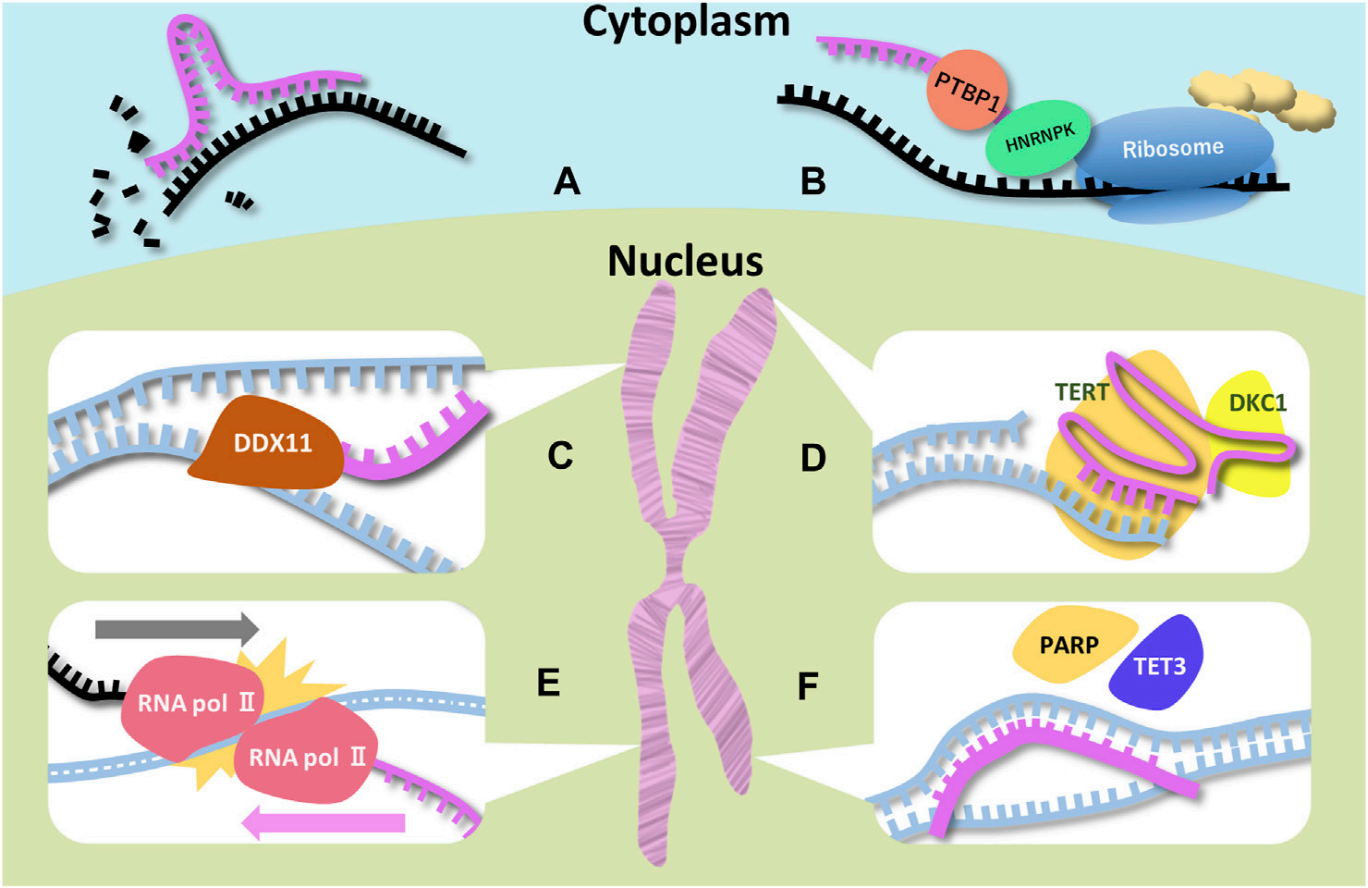
Schematic representations of function of ncRNAs and RBPs associated with microcephaly Six examples for ncRNAs (miRNA, SINEUP, DDX11-AS1 (CONCR), TERC, UBE3A-ATS, and pancRNA) are illustrated. Magenta, black, and blue strands indicate ncRNA, mRNA, and DNA respectively. **(A)** Degradation of the target mRNA is a miRNA function. MiRNAs have the complementary sequence of the target mRNAs. **(B)** Two RBPs (PTBP1 and HNRNPK) bind to SINEUP and recruit the ribosome. SINEUP upregulates translation through RBPs. **(C)** DDX-AS1 bind to DDX11 directly to promote its enzymatic activity. In addition, the lncRNA traps miRNA targeting DDX11 mRNA. **(D)** TERC is the template for telomere elongation. DKC1 is essential for the TERC stability. **(E)** RNA polymerases colliding is thought to lead to stopping elongation of UBE3A mRNA. **(F)** pancRNA recruits transcription and histone acetylation factors by changing DNA structure.

Abnormal brain growth leads to aberrant brain size and developmental disorders. Microcephaly is defined as a head circumference < −2 standard deviations (SD) in humans (Whelan, 2010). Genetic mutations have been identified in half of such patients. Patients with severe microcephaly (<-3 SD) are more likely to be also have other developmental diseases such as epilepsy, cerebral palsy, autism, and intellectual disabilities simultaneously (Pirozzi et al., 2018). Numerous studies have revealed a variety of risk genes for microcephaly. For example, the *assembly factor for spindle microtubules (ASPM)* gene, which encodes a centrosomal protein, is one of the most frequent candidate genes for this symptom (Nicholas et al., 2009). Dysfunction of other centrosomal proteins such as WDR62, CEP135, CENPE, and MCHP1 also causes microcephaly, which indicates the importance of centrosomes for brain volume expansion in infants (Pirozzi et al., 2018). On the other hand, non-genetic factors (e.g., Zika virus infection, excessive maternal alcohol drinking, drug overdose, and malnutrition) can also be causes for such diseases (Whelan, 2010). In addition, epigenetic factors are known to be involved in abnormal brain growth phenotypes. For example, Rett syndrome, an epigenetic disease, was first described in 1966 (Rett, 1966). The syndrome appears in approximately 1 in 10,000 female births. Patients grow and develop normally until 6-8 months of age, and then gradually lose speech and hand skills and appear to have stereotypic hand movements. The head circumference growth decelerates and patients are diagnosed with microcephaly (Weng et al., 2011). This disorder is caused by mutation in *X-linked methyl-CpG-binding protein 2 (MeCP2)*, whose protein product binds to methyl-CpG sites (Amir et al., 1999), affecting both genic and intergenic regions in the genome to modulate RNA transcription. The occurrence of the complex disease phenotypes is further supported by recent studies showing that many central nervous system (CNS) disorders are also associated with mutations in non-coding regions in the human genome (Simon-Sanchez and Singleton, 2008). Single nucleotide polymorphisms (SNPs) located within non-coding regions have occasionally been found in infants with microcephaly (Xia et al., 2017). A recent study indicated that ASPM is modulated by circular RNA and microRNA (miRNA), both of which are types of non-coding RNAs (ncRNAs) (Han et al., 2021). Therefore, better understanding of the involvement of non-coding regions in the pathogenesis of microcephaly through ncRNA transcription is needed. NcRNAs play important roles in genome transcription, RNA translation, RNA degradation, and protein scaffolding (Yan et al., 2021). For example, several miRNAs related to Feingold syndrome function in RNA interference in which the precursors of these miRNAs are transcribed by RNA polymerase Ⅱ, and then the miRNA is incorporated into the miRNA-induced silencing complex called miRISC to degrade the target mRNA (de Pontual et al., 2011), as described later. In addition to miRNA, long ncRNA (lncRNA) with size greater than 200 nt (Novikova et al., 2013) also seems to function for regulating brain size by forming a complex structure with chromatinic DNA to regulate gene expression (Chi et al., 2019). Here, we will introduce diseases with microcephaly candidate genes including those for RNA-binding proteins (RBP) and with intergenic mutations that affect the generation of ncRNAs and discuss how ncRNAs are involved in establishing the nature of human-specific “large-brain” and how RNA-involving epigenetic mechanisms can be therapeutic targets (Figure 1). In fact, there are several brain diseases that affect brain size but are not annotated as microcephaly. Since little information on ncRNAs contributing to microcephaly is available, we will also refer to ncRNAs known to be physically and/or functionally connected to brain-size-affecting diseases (e.g., autism spectrum disorder: ASD) other than known microcephaly-related diseases.

## 2 Cytosolic function of ncRNAs in brain diseases

### 2.1 Microcephaly-related inhibitory ncRNAs in the cytoplasm

Feingold syndrome is an autosomal dominant syndrome including microcephaly, short stature, and short mesophalanx of the fifth finger (brachymesophalangy). Several ncRNAs are involved in the pathogenesis of this syndrome. In many cases, the deletion of either *MYCN* (type 1) or *MIR17HG* (type 2) seems to cause this type of disease (de Pontual et al., 2011). *MIR17HG* generates six miRNAs, namely, miR-17, 18a, 19a, 20a, 19b-1, and 92a-1 (Mendell, 2008), which have been reported to be involved in proliferation of various tumors (Tan et al., 2022). MYCN protein seems to regulate the expression of these miRNAs by binding to the *MIR17HG* promoter region to upregulate miRNA expression (de Pontual et al., 2011). MiR-17-92 cluster is described as a human oncogene in several cancers (Hayashita et al., 2005) (Mu et al., 2009). The deletion of the cluster promotes apoptosis because the miRNAs target *BIM* (Ventura et al., 2008), which initiates the intrinsic apoptotic pathway (Sionov et al., 2015). Mice models for Feingold syndrome type 2 exhibit brachymesophalangy, small body (short stature), and microcephaly. The homozygous deletion of *MIR17HG* frequently leads to perinatal lethality (de Pontual et al., 2011). *MIR17HG* targets *TGF-β receptor type 2 (TGFBR2)* (Ma et al., 2016) (Mirzamohammadi et al., 2018), and cases deficient for *MIR17HG* are associated with excessive TGF-*β* signaling, which is supported by the fact that treatment with a TGF-*β* receptor inhibitor, LY364947, prevented the skeletal defect and microcephaly in the Feingold syndrome type 2 mouse model. GW788388, another TGF-*β* receptor inhibitor, and 1D11, a neutralizing antibody against TGF-*β* ligands, also caused similar effects (Mirzamohammadi et al., 2018).

### 2.2 Gene-activating ncRNAs functioning in the cytoplasm

In recent years, patients with *de novo* mutation of *RAB11B* have been described. The symptoms include absent speech, epilepsy, hypotonia, and microcephaly (Lamers et al., 2017) (Jauss et al., 2022), in spite of the fact that, in mouse, *Rab11b* deficiency exhibits no phenotypes (Nassari et al., 2020). This suggests that human *RAB11B* has acquired human-specific functions. RAB11B is a small GTPase belonging to the Rab family. Rab forms and transfers vesicles, and fuses them with the cellular membrane (Stenmark and Olkkonen, 2001). *RAB11B* is expressed in the brain, heart, and testis (Lai et al., 1994). Mislocalization of abnormal RAB11B due to mutations at its GTP/GDP binding pocket causes disorganized brain structures and functions (Lamers et al., 2017). Interestingly, *RAB11B-AS1* is transcribed from the bidirectional *RAB11B* promoter to modulate *RAB11B* functions. *RAB11B-AS1* is expressed in humans including in the brain, and functions as a “SINEUP” RNA for *RAB11B*, that can promote *RAB11B* translation (Zarantonello et al., 2021). SINEUP is a category of lncRNAs that promote translation of partly overlapping mRNAs (Zucchelli et al., 2015). This mechanism involves Polypyrimidine tract-binding protein (PTBP1), which is also known to function in alternative splicing for *Filamin A (FLNA)* (Zhang et al., 2016), a causative gene for microcephaly in mice, and the deregulation of this alterative splicing leads to periventricular heterotopia (PH) in human (Lian et al., 2012). In addition, PTBP1 can function together with heterogeneous nuclear ribonucleoprotein K (HNRNPK) to bind to the SINEUP RNAs to target mRNAs. These two RBPs help to recruit ribosomal subunits for enhancing the translation of the target mRNAs (Toki et al., 2020). It is noteworthy that *RAB11B* and *RAB11B-AS1* are downregulated by *CDH8* (Zarantonello et al., 2021), and therefore, known *CDH8* variants may modulate the cellular level of *RAB11B*, leading to macrocephaly and intellectual disability (Bernier et al., 2014). Although the molecular function of *RAB11B-AS1* in brain contexts is still obscure, it is possible that evolutionary acquisition of *RAB11B-AS1* was actively involved in enlarging the human brain because some studies have shown an association with cancer via oncogene effects such as cell proliferation (Li et al., 2020), migration and invasion (Niu et al., 2020).

## 3 Epigenetic function of ncRNAs in brain disease

### 3.1 Chromatinic ncRNAs acting on intergenic regions

Warsaw breakage syndrome is a recessive hereditary disease caused by mutation in *DEAD/H-box helicase 11* (*DDX11*, also known as *hChlR1*) (van der Lelij et al., 2010). The clinical features include microcephaly, hearing loss, and facial dysmorphia. DDX11 regulates chromatin structure (Pisani et al., 2018). *DDX11* also controls chromosome separation and sister chromatid condensation in mitosis. *DDX11* is hypothesized to prevent abnormal DNA structure in the replication folk (Leman et al., 2010). In line with this idea, defective sister chromatid cohesion is frequently observed in Warsaw breakage syndrome patients’ cells (Pisani, 2019). Separation of the centromere and sister chromatid pairs observed in mitomycin C-induced chromosomal breakage is a remarkable feature of *DDX11* mutations (van der Lelij et al., 2010). The mutations in conserved helicase motifs result in unwinding forked duplex DNA substrates. DDX11 destabilization occurs due to misfolding of the protein (Santos et al., 2021). In mouse models, *Ddx11* is indispensable for mouse embryonic and placental development, and *Ddx11* knock out causes embryonic lethality (Inoue et al., 2007). In zebrafish models, embryonic lethality was increased and craniofacial and vertebral abnormalities were observed. In addition, *ddx11* dysfunction generated heterochromatic structures ectopically. This gene also affects histone epigenetic modifications (Sun et al., 2015). Interestingly, a lncRNA, *DDX11 antisense RNA 1* (DDX11-AS1, also known as *CONCR*) is transcribed bidirectionally from the *DDX11* promoter region. Although the molecular function of *DDX11-AS1* in microcephaly contexts is still obscure, deletion of *DDX11-AS1* causes a defect in sister chromatid condensation in mitosis like Warsaw breakage syndrome. Unlike SINEUP, the DDX11 protein level is not affected by the ncRNA knockdown. Levels of histone H3K9 acetylation at the *DDX11* promoter region and *DDX11* mRNA are also unchanged. Surprisingly, however, the ncRNA can bind DDX11 protein directly, and thus activates hydrolysis of ATP. *DDX11-AS1* maintains proper chromatin structure through promoting the enzymatic activity of DDX11 (Marchese et al., 2016). Another report indicated that *DDX11-AS1* also function to regulate *DDX11* through sponging miR-873-5p, which can target *DDX11* (Zhang et al., 2020).

### 3.2 ncRNA that maintains telomere length

Mutation of *dyskerin pseudouridine synthase 1 (DKC1)* frequently result in Hoyeraal-Hreidarsson syndrome, a microcephaly disease (Dehmel et al., 2016). DKC1 has TRUB (tRNA pseudouridine synthase B-like) and PUA (pseudouridine synthase and archaeosine transglycosylase) domains. The TRUB domain constitutes the catalytic core of DKC1, whereas the PUA domain seems to function as a RNA binding motif (Garus and Chantal, 2021). *DKC1* plays an important role in pseudouridylation of rRNA and telomere extension. The telomerase complex is composed of telomerase reverse transcriptase (TERT), telomerase RNA component (TERC), and other protein factors including DKC1 (Czekay and Kothe, 2021). Apoptosis and chromosomal aberrations increase and proliferative potential decreases in *Terc* ^*−/−*^ mouse cells (Wong et al., 2003). Both Hoyeraal-Hreidarsson syndrome patients and mouse models for DKC1 dysfunction show reduced rRNA processing and telomerase activities (Mochizuki et al., 2004). In fact, loss of telomere length cause dyskeratosis congenita characterized by bone marrow failure, hyperpigmentation, nail dystrophy and leukoplakia (AlSabbagh, 2020). In particular, *DKC1* is involved in the Xp28 and X-linked recessive dyskeratosis congenita, known as a profound type of dyskeratosis congenita, including growth retardation and microcephaly (Dehmel et al., 2016). In most cases of Hoyeraal-Hreidarsson syndrome, the variant A353V located in the PUA domain of DKC1 is observed (Knight et al., 1999). The same mutation attenuates the binding of DKC1 to the TERC, leading to TERC destabilization (Czekay and Kothe, 2021). Accordingly, some patients with mild dyskeratosis congenita also have telomere shortening (Vulliamy et al., 2001) (Yamaguchi et al., 2003). Because bone marrow failure also accompanies dyskeratosis congenita, the patients are frequently treated with hematopoietic cell transplantation (HCT) or androgen therapy (Savage and Niewisch, 2009). Considering the potential of ncRNAs as future therapeutic agents for curing such diseases, their physical association with EXOSC10, a component of the RNA exosome complex which eliminates TERC, may be notable, because its knockdown restored telomerase activity in *DKC1* knockdown cells (Shukla et al., 2016).

### 3.3 The convergent regulation of gene expression by ncRNA

Angelman syndrome, another microcephaly disease, was first described in 1965. It was characterized by unusual arm position and jerky movement (Kishino et al., 1997). Major characteristics include severe intellectual disability, lack of speech, sleep disruption, and microcephaly (Levin et al., 2022). Mouse models for Angelman syndrome frequently exhibit motor dysfunction and deficits in learning and memory. Abnormal electroencephalogram (EEG) is also observed (Miura et al., 2002). Mutation in the *E6-AP ubiquitin-protein ligase gene (UBE3A)* was found in chromosome 15 of many patients (Kishino et al., 1997) (Matsuura et al., 1997). Normally, *UBE3A* is expressed only from the maternal allele in the brain, while the paternal allele is silenced by genome imprinting. Some patients have a UBE3A mutation in the maternal allele, and others have paternal uniparental disomy (PUD) and/or imprinting defects (ID) (Saitoh et al., 2005) (Bai et al., 2014). The deletion patients have more profound effects than PUD and ID patients (Lossie et al., 2001). A *UBE3A* antisense transcript, *UBE3A-ATS*, suppresses *UBE3A* on the paternal chromosome (Meng et al., 2012). In the paternal chromosome, *UBE3A* and *UBE3A-ATS* are transcribed at the same time. However, in contrast to SINEUP, *UBE3A-ATS* prevents *UBE3A* transcription at the expressing paternal allele. It has been thought that 2 opposing RNA polymerases collide and stop the elongation of *UBE3A* mRNA (Wang et al., 2021) (Mabb et al., 2011). Although the molecular function of *UBE3A-ATS* in microcephaly contexts is still obscure, disrupting *UBE3A-ATS* transcription is noted as a potential therapy to increase *UBE3A* expression in the gene therapy field. For example, a clinical trial using antisense oligonucleotides is ongoing (Schmid et al., 2021). In a mouse model, such treatment can recover paternal *UBE3A* expression. Early treatment in mouse models (at postnatal day 1) is more effective compared with treatment of the adult (at 2 to 4 months of age). Partial improvement of motor deficiency and anxiety is observed only in young models. However, the behavioral phenotypes are hardly recovered. Nonetheless, both early and adult treatments ameliorate the memory impairment in fear conditioning tests (Milazzo et al., 2021) (Meng et al., 2015). Creation of indels located between the *Ube3a* 3’ UTR and *Snord115 (Small Nucleolar RNA, C/D Box 115)* by CRISPR/Cas9 rescued the behavioral phenotype (Schmid et al., 2021). Cas9 targeting the *Snord115* cluster also prevent the motor deficiency (Wolter et al., 2020). Injection of the adeno-associated virus (AAV) expressing Zinc finger-based artificial transcription factors (ATFs), that repress *Ube3a-ats* to induce *Ube3a* expression (Bailus et al., 2016), also recovers the behavioral phenotype in mouse models (O'Geen et al., 2023). It is noteworthy that these targetings simultaneously truncate *UBE3A-ATS*, supporting the idea that allele-specific artificial removal of *UBE3A-ATS* is essential for ongoing therapies. How the lncRNA represses *UBE3A* expression is still uncertain, and elucidation of the mechanism will enable more effective therapy for Angelman syndrome.

### 3.4 ncRNAs leading to divergent transcription

**FIGURE 2:**
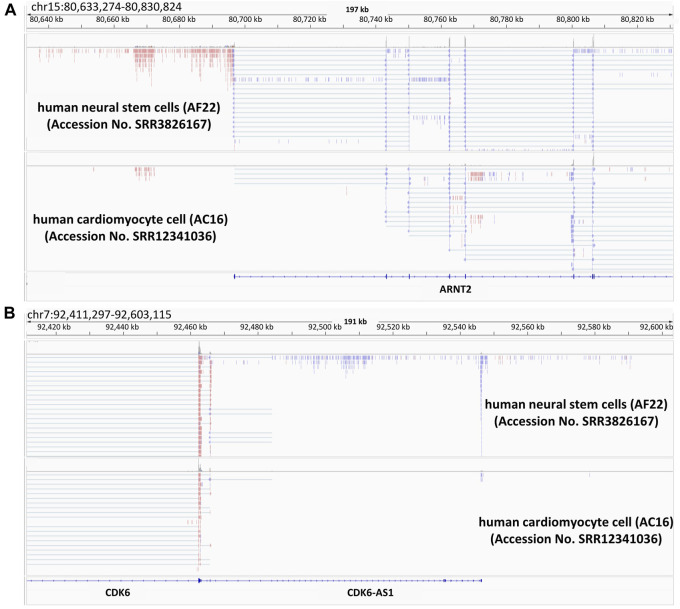
Presence of divergent lncRNAs in two examples of microcephaly-related genes Snapshots of the Integrative Genomics Viewer. Publicly available RNA-seq data of human-iPS cell-derived neural stem cell (AF22) and human hybrid cardiomyocyte (AC16) are shown. The data is from (Brattas et al., 2017; Lopacinski et al., 2021). ARNT2 **(A)** and CDK6 **(B)** are microcephaly related genes. In this figure, the colors indicate the differential strand usage.

We have illuminated various points of ncRNA actions in the above sections. Independently from convergent lncRNAs, we have found a different class composed of thousands of lncRNAs resulting from divergent transcription that originates from protein-coding gene promoters (Uesaka et al., 2014). Later on, we will introduce the functional mode and the potentials of such divergent ncRNAs based on our previous and other studies. As shown in the upper panel of Figures 2A lncRNA seems to be transcribed in the reverse direction to the partner gene. Comparison between RNA-seq reads from human neural stem cells (Brattas et al., 2017) and those from human cardiomyocyte cells (Lopacinski et al., 2021), revealed that *aryl hydrocarbon receptor nuclear translocator 2 (ARNT2)* is more highly expressed in brain than in cardiomyocytes. A variant of the gene causes Webb-Dattani syndrome, of which the features include microcephaly (Webb et al., 2013). Likewise, *cyclin dependent kinase 6 (CDK6)* is also microcephaly candidate gene (Naveed et al., 2018) and lncRNA expression was synchronized with that of mRNA. It would be interesting to see the possible effects of divergent lncRNAs on the pathogenesis of microcephaly-related diseases. As noted above, we have discovered a new type of divergent lncRNAs, called promoter-associated lncRNAs (pancRNAs) that are transcribed in the reverse direction to a set of tissue-specific genes (Uesaka et al., 2017). Approximately half of mammalian promoters show CpG-rich sequences and lack of TATA elements. In these CpG island-type promoters without TATA elements, TATA-binding protein (TBP) is recruited together with CpG-rich sequence-specific transcription factors such as Sp1 (Wu and Sharp, 2013) in both strands, thereby driving bidirectional transcription (Mahpour et al., 2018). Although enormous numbers of genes, including housekeeping genes, have CpG-rich promoters, the characteristics of the promoters for pancRNA-partnered genes include the acquisition of a G- and/or C-skewed motif, while such a skew cannot be seen in housekeeping genes. In addition, the lack of a poly(A) site sequence in the body of the pancRNAs has enabled then to get longer (An et al., 2021). Promoter-proximal Ser2 phosphorylation further reinforces a longer RNAPII dwell time at the start site, which may be beneficial for recruiting U1 snRNP upstream of the gene, thereby suppressing the recognition of poly(A) sites and the coupled termination of divergent transcription (Almada et al., 2013). In line with the concordant expression of pancRNAs and the partnered genes, as shown in Figures 2A,B, pancRNA production is associated with DNA demethylation, H3K4 trimethylation (Hamazaki et al., 2015), and H3K27 acetylation (Uesaka et al., 2017). In terms of the biological functions of pancRNAs, these are dependent on the roles of the downstream genes. For example, in rat PC12 cells, *pancNusap1* functions in *nucleolar and spindle associated protein 1 (Nusap1)* activation through histone acetylation, accelerating the cell cycle since Nusap1 plays a role in spindle microtubule organization (Yamamoto et al., 2016). Another example is mouse *pancIl17d*, which enhances demethylation of the *interleukin 17days (Il17d)* promoter by recruiting ten-eleven translocation 3 (Tet3) and poly ADP-ribose polymerase (Parp). Silencing *pancIl17d* is embryonic lethal, probably because Il17d functions to support proliferation/differentiation of pluripotent stem cells, which has been evidenced by the fact that supplying Il17d protein rescues embryonic survival (Hamazaki et al., 2015). pancRNAs occasionally form a triple helix structure with the DNA duplex of promoters and/or enhancers, and interact with some regulatory proteins, such as histone modifiers and transcription factors, to regulate gene transcription in cis. A second mechanism is based on transcriptional activation via formation of a DNA-RNA hybrid (R-loop). In mammalian cells, the asymmetrical distribution of cytosine and guanine, one of the characteristics of CpG islands for tissue-specific genes as discussed above, makes it easy to form R-loops (Chen et al., 2017). Therefore, targeting these structures triggered by pancRNA expression might be a strategy to mitigate microcephaly-related diseases in the future.

## 4 RBPs as potential targets for brain diseases

Although the information on ncRNAs in microcephaly is still limited, we can learn more about ncRNAs in relation to brain diseases. In addition to the examples of functional ncRNAs noted above, several other ncRNAs that specify social interactions and behavior have been identified by using mouse models (Table 1). For example, brain cytoplasmic 1 (BC1), which has a motif for dendritic localization (Robeck et al., 2016), regulates neuronal activity-dependent translation in neurons (Eom et al., 2014). Memory and learning dysfunction were observed in some mouse knockout models of BC1 ncRNAs (Chung et al., 2017). The lncRNA nuclear paraspeckle assembly transcript 1 (Neat1) sponges various miRNAs (Azizidoost et al., 2022). The knockout model of Neat1 lost interest in a social interaction (Kukharsky et al., 2020). The ncRNA of synapsin2 (syn2) is decreased in mice with dominant behavior. The ncRNA modulates the social rank thorough binding syn2b pre-mRNA directly and protecting against its destabilization (Ma et al., 2020). In ASD and schizophrenia (SCZ), differentially expressed lncRNAs were detected (Ziats and Rennert, 2013) (Chen et al., 2016) (Table 2). One can hypothesize that most of the lncRNAs function together with RBPs. Although we still do not know of RBPs specifically functioning in the context of microcephaly, in some cases of brain diseases, detailed relationships between RBPs and ncRNAs have been revealed. Cyrano (OIP5-AS1), which is a schizophrenia candidate gene in females (Safari et al., 2019), sponges HuR (human antigen R) and inhibits the protein (Kim et al., 2016). Gomafu (RNCR2/MIAT) binds to the RNA-binding protein Celf3 and splicing factor SF1. The complex is speculated to control splicing and transcription (Ishizuka et al., 2014). TUNA (Tcl1 Upstream Neuron-Associated lincRNA) forms an RNA-RBP complex with three RBPs, PTBP1, HNRNPK, and nucleolin (NCL), and the complex binds to the *sox2* promoter (Lin et al., 2014). Rhabdomyosarcoma 2-associated transcript (RMST) and SOX2 interaction plays an important role in neural stem cell fate specification (Ng et al., 2013). A recent study has shown that lncRNAs determine Sox2’s genomic localization (Hamilton et al., 2023). In another example, the interaction of the transcription factor POU3F3 and DNMT1-associated long intergenic (Dali) was described (Chalei et al., 2014). We believe that accumulating evidence further opens up the possibility of lncRNAs as therapeutic targets to artificially regulate their association with various RBPs.

**TABLE 1:** NcRNA-involving phenotypes in mouse model.

Name	Phenotype	Ref
BC1	Learning and memory impaired	Chung et al. (2017)
Neat1	Determination of behavioral responses under conditions of stress	Kukharsky et al. (2020)
AtLAS	Regulation of social hierarchy	Ma et al. (2020)
Linc-Brn1b	Generation of upper layer II-IV neurons in the neocortex	Sauvageau et al. (2013)
Pnky (lnc-pou3f2)	Neuronal differentiation	Ramos et al. (2015)
Malat1	Synapse formation and/or maintenance	Bernard et al. (2010)
GM12371	Regulation of expression of synaptic gene	Raveendra et al. (2018)
Bdnf-AS	Maintenance of stemness in neural stem cells	Modarresi et al. (2012)
Evf2	Formation of GABA-dependent neuronal circuitry	Bond et al. (2009)
Dali	Regulation of neural differentiation genes	Chalei et al. (2014)
Zfas1	Upregulating in status epilepticus mice model	Hu et al. (2020)
Dlx6-as1	Upregulating in Parkinson’s disease (PD) mice model	Liu et al. (2022)

**TABLE 2:** NcRNAs related to human brain diseases.

ncRNA	Disease	Ref
BC200	Alzheimer’s disease (AD)	Sosinska et al. (2015)
NEAT1	Amyotrophic lateral sclerosis (ALS), Epilepsy, SCZ (female), AD, PD	Safari et al. (2019); An et al. (2020)
BDNF-AS	ASD	Wang et al. (2015)
MSNP1AS	ASD	Kerin et al. (2012)
DGCR5	SCZ	Meng et al. (2018)
RP5-998N21.4	SCZ	Guo et al. (2022)
DODA-AS1 (G30)	SCZ, bipolar disorder	Detera-Wadleigh and McMahon (2006)
Cyrano (OIP5-AS1)	SCZ (female)	Safari et al. (2019)
FAS-AS1	SCZ (male)	Safari et al. (2019)
Gomafu (MIAT/RNCR2)	Multiple sclerosis (MS), SCZ	Barry et al. (2014); Fenoglio et al. (2018)
TUNA	Huntington’s Disease (HD)	Lin et al. (2014)
RMST	PD	Chen et al. (2022)
PTCHD1-AS	ASD	Ross et al. (2020)
MEG3	ASD	Taheri et al. (2021)

## 5 Conclusion

In this review, we described five ncRNAs that regulate microcephaly-related genes. Although little information is available on ncRNAs responsible for microcephaly, multiple factors are known to provoke microcephaly. For example, 30% of case of ASD are accompanied by the features of diminishing brain size (Fombonne et al., 1999). Table 2 shows the ncRNAs known to be related to brain diseases. In fact, there are many ncRNAs related to ASD. Interestingly, most of the ncRNAs in this list are categorized as lncRNA species. Therefore, it would be interesting to confirm whether the lncRNA class rather than the small RNA class brain function tends to affect the determination of brain size by analyzing the lncRNAs listed in Table 2. Along with understanding of the human genome, tailor-made medicine is a center of attention these days. Acquisition of the sequences of individual genomes become easier and less expensive, revealing mutations that occur not only in coding genes but also in intergenic regions. In particular, accessible and affordable sequence reading enables us to find new intergenic mutations that could have been missed previously because of mild disease symptoms and poor sequencing technology. The resultant studies on intergenic regions allow us to highlight the potentials of ncRNAs for understanding human pathology in clinical research. Since the intergenic regions are poorly conserved among the enormous variety of organisms, and the large size and complicated functions of the brain are human-unique features, it is intriguing possibility that the intergenic regions contribute a big controlling center for determining such interesting traits. Considering the human-specific features of the brain structure and function, it seems likely that model animals such as mouse, zebra fish, and fruit fly would be of limited use for searching for human-specific ncRNAs. Leveraging human brain organoids, genome-wide association studies (GWASs), and massive annotation of human-specific ncRNA functions are essential for pioneering this vast ncRNA field. This field will lead us to new treatments for brain disease and understanding what makes us human.
